# Supplementary material for: A SUMO-interacting motif in the guanine nucleotide exchange factor EPAC1 is required for subcellular targeting and function
Source: J Biol Chem. 2025 May 22;301(6):110279. doi: 10.1016/j.jbc.2025.110279 (PMC12226364; doi:10.1016/j.jbc.2025.110279)
Supplement: Supplementary Figures [file mmc1.docx]

**A SUMO-interacting motif in the guanine nucleotide exchange factor EPAC1 is required for subcellular targeting and function**

Wenli Yang^1,2^, Fang Mei^1,2^, Wei Lin^1,2^, Jason E. Lee^3^, Si Nie^4^, Christopher J. Bley^4^, André Hoelz^4,5^, and Xiaodong Cheng^1,2,^*

^1^Department of Integrative Biology and Pharmacology, The University of Texas Health Science Center, Houston, Texas, USA.

^2^Texas Therapeutics Institute, The Brown Foundation Institute of Molecular Medicine for the Prevention of Human Diseases, The University of Texas Health Science Center, Houston, Texas, USA.

^3^Department of Molecular and Cellular Biology, Baylor College of Medicine, Houston, Texas, USA

^4^Division of Chemistry and Chemical Engineering, California Institute of Technology, Pasadena, California, USA.

^5^Howard Hughes Medical Institute, California Institute of Technology, Pasadena, California, USA.

* Xiaodong Cheng

**Email:**  xiaodong.cheng@uth.tmc.edu

**Keywords:** cAMP; SUMOylation; nuclear pore complex; nuclear envelope; RanBP2/Nup358.

**Supplementary Figures**

**
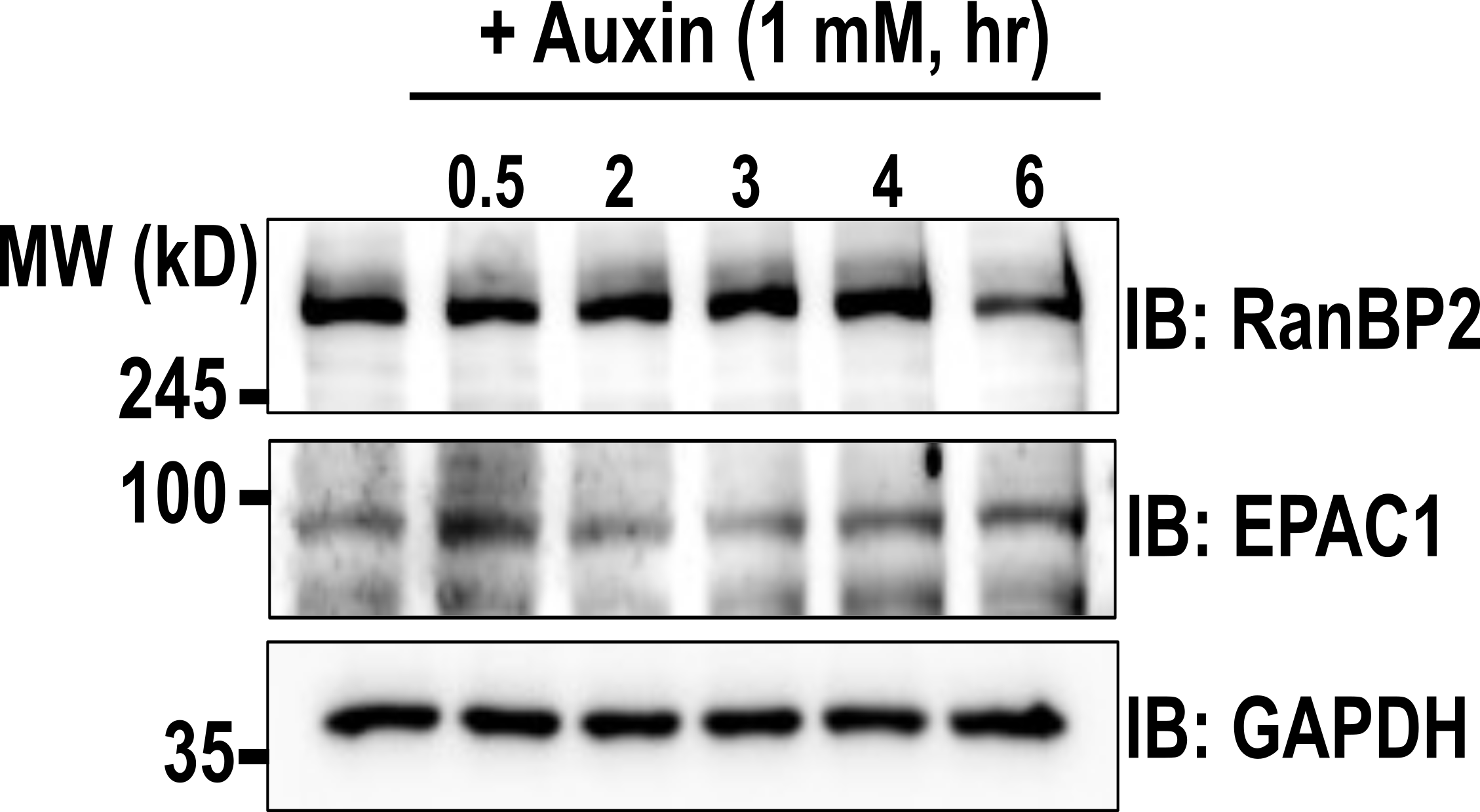
**

**Figure S1. Effects of Auxin treatment on endogenous cellular RanBP2 and EPAC1 in HCT116 cells.** The cellular levels of RanBP2, EPAC1, and GAPDH probed by immunoblotting analysis in HCT116 cells treated with 1 mM auxin at 37 °C for various times.

**
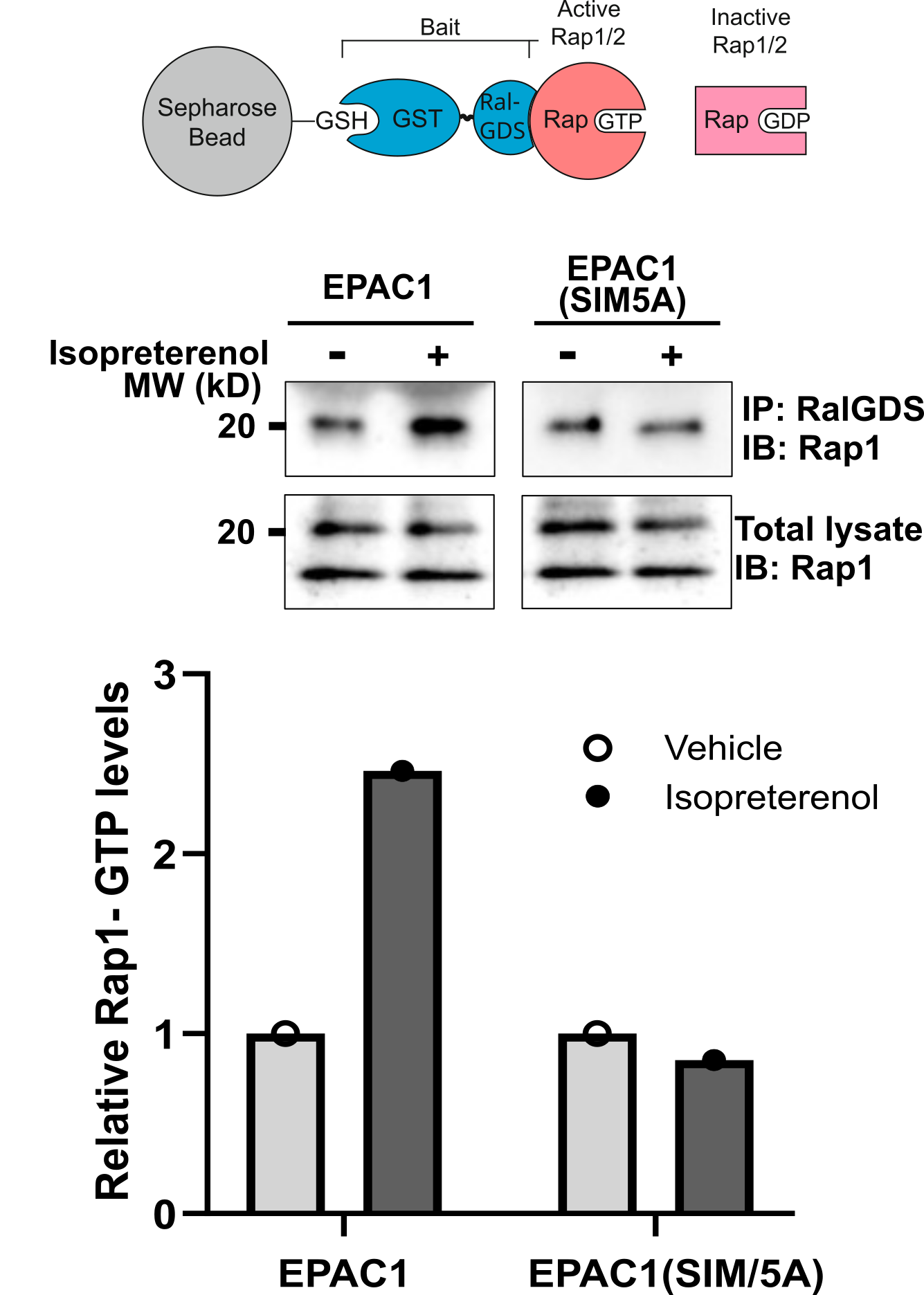
**

**Figure S2. EPAC1 SIM mutation interferes with cAMP**-**induced cellular activation of small GTPase Rap1.** Levels of cellular Rap1-GTP in HEK293 cells ectopically expressing EPAC1-EYFP or EPAC1(SIM5A)-EYFP in response to 20 µM isoproterenol treatment for 30 min.

**
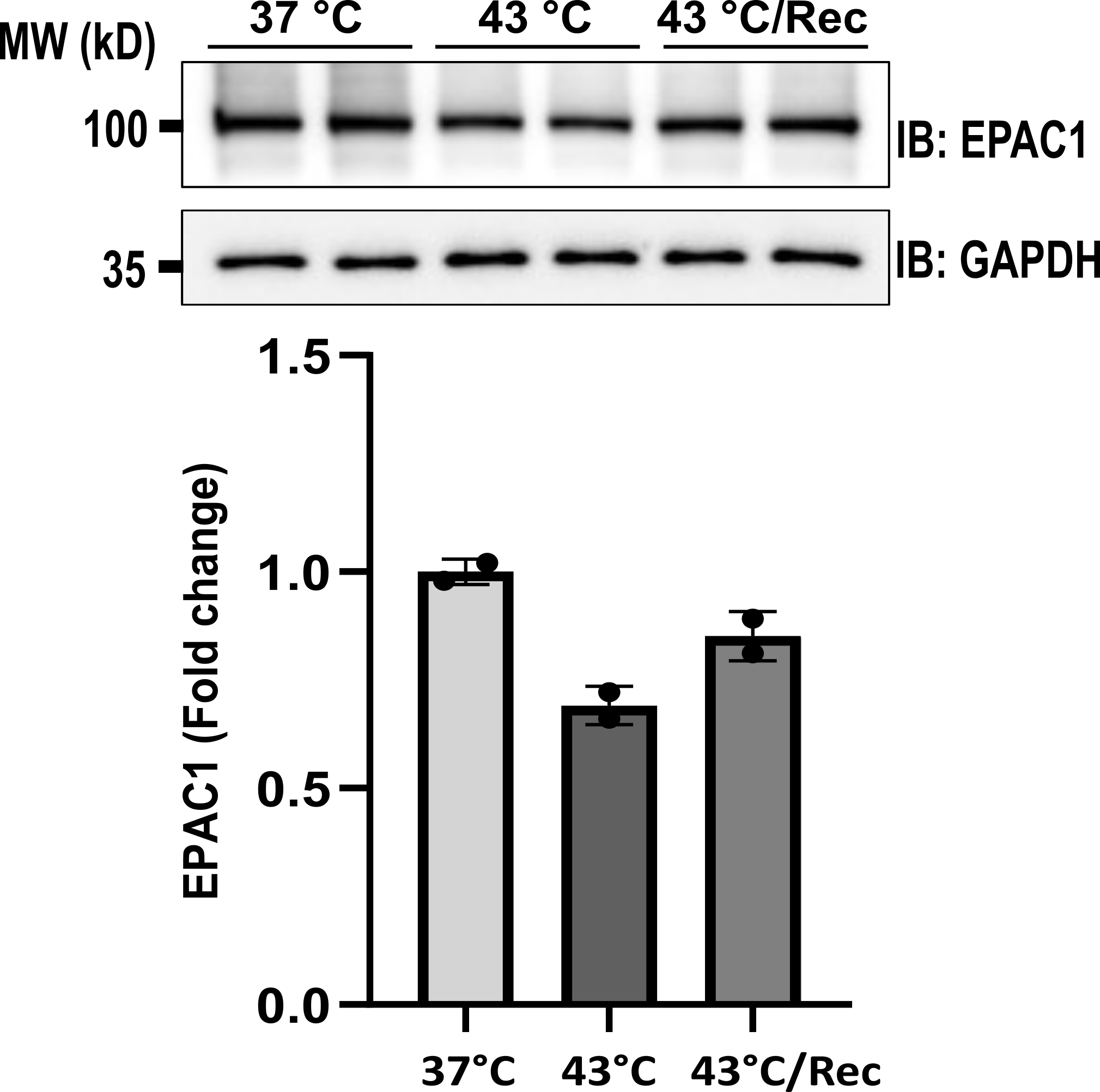
**

**Figure S3. Effect of heat shock on cellular EPAC1.** Levels of cellular EPAC1-APEX2 in HEK293 cells ectopically expressing EPAC1- APEX2 in response to heat shock (43 °C, 30 min) and heat shock plus recovery (37 °C, 2h).
